# The Genographic Project Public Participation Mitochondrial DNA Database

**DOI:** 10.1371/journal.pgen.0030104

**Published:** 2007-06-29

**Authors:** Doron M Behar, Saharon Rosset, Jason Blue-Smith, Oleg Balanovsky, Shay Tzur, David Comas, R. John Mitchell, Lluis Quintana-Murci, Chris Tyler-Smith, R. Spencer Wells, The Genographic Consortium

**Affiliations:** 1 Genomics Research Center, Family Tree DNA, Houston, Texas, United States of America; 2 Molecular Medicine Laboratory, Rambam Health Care Campus, Haifa, Israel; 3 Data Analytics Research Group, IBM T. J. Watson Research Center, Yorktown Heights, New York, United States of America; 4 The Genographic Project, National Geographic Society, Washington, District of Columbia, United States of America; 5 Research Centre for Medical Genetics, Russian Academy of Medical Sciences, Moscow, Russia; 6 Unitat de Biologia Evolutiva, Universitat Pompeu Fabra, Barcelona, Spain; 7 Department of Genetics, La Trobe University, Bundoora, Australia; 8 Institut Pasteur, Paris, France; 9 CNRS, URA3012, Paris, France; 10 The Wellcome Trust Sanger Institute, Wellcome Trust Genome Campus, Hinxton, Cambridge, United Kingdom; University of Oxford, United Kingdom

## Abstract

The Genographic Project is studying the genetic signatures of ancient human migrations and creating an open-source research database. It allows members of the public to participate in a real-time anthropological genetics study by submitting personal samples for analysis and donating the genetic results to the database. We report our experience from the first 18 months of public participation in the Genographic Project, during which we have created the largest standardized human mitochondrial DNA (mtDNA) database ever collected, comprising 78,590 genotypes. Here, we detail our genotyping and quality assurance protocols including direct sequencing of the mtDNA HVS-I, genotyping of 22 coding-region SNPs, and a series of computational quality checks based on phylogenetic principles. This database is very informative with respect to mtDNA phylogeny and mutational dynamics, and its size allows us to develop a nearest neighbor–based methodology for mtDNA haplogroup prediction based on HVS-I motifs that is superior to classic rule-based approaches. We make available to the scientific community and general public two new resources: a periodically updated database comprising all data donated by participants, and the nearest neighbor haplogroup prediction tool.

## Author Summary

The Genographic Project was launched in 2005 to address anthropological questions on a global scale using genetics as a tool. Samples are collected in two ways. First, the project comprises a consortium of ten scientific teams from around the world united by a core ethical and scientific framework that is responsible for sample collection and analysis in their respective region. Second, the project promotes public participation in countries around the world and anyone can participate by purchasing a participation kit (Video S1). The mitochondrial DNA (mtDNA), typed in female participants, is inherited from the mother without recombining, being particularly informative with respect to maternal ancestry. Over the first 18 months of public participation in the project we have built up the largest to date database of mtDNA variants, containing 78,590 entries from around the world. Here, we describe the procedures used to generate, manage, and analyze the genetic data, and the first insights from them. We can understand new aspects of the structure of the mtDNA tree and develop much better ways of classifying mtDNA. We therefore now release this dataset and the new methods we have developed, and will continue to update them as more people join the Genographic Project.

## Introduction

The plethora of human mitochondrial DNA (mtDNA) studies in recent years has made this molecule one of the most extensively investigated genetic systems. Its abundance in human cells; uniparental, nonrecombining mode of inheritance; and high mutation rate compared to that of the nuclear genome, has made mtDNA attractive to scientists from many disciplines. Knowledge of mtDNA sequence variation is rapidly accumulating, and the field of anthropological genetics, which initially made use of only the first hypervariable segment (HVS-I) of mtDNA, is currently being transformed by complete mtDNA genome analysis [1]. While contemporary combined sources offers approximately 65,000 HVS-I records (Oleg Balanovsky, unpublished data) and over 2,000 complete mtDNA sequences, difficulties remain in standardizing these published data, as they report varying sequence lengths and different coding-region SNPs, and apply any number of methodologies for classifying haplotypes into informative haplogroups (Hgs) [2,3]. For example, some studies have defined the HVS-I range to comprise nucleotides 16093–16383 [4], some 16024–16365 [5], some adhered to the widely accepted definition of 16024–16383 [6], while others extended the reported range to include positions such as 16390 and 16391 due to their predictive value in identifying certain specific clades [7,8]. Even more serious is the problem of Hg assignment, which, in the absence of complete sequence data, is best achieved by genotyping a combination of coding-region biallelic polymorphisms. Forensic studies (which comprise a significant portion of the existing dataset) and many population studies published before 2002 have predicted Hgs based on the HVS-I motif alone, thereby ignoring the occurrence of homoplasy and back mutations [2,9]. Moreover, it has been shown that many published mtDNA databases contain errors that distort phylogenetic and medical conclusions [10–15]. Therefore, it has become abundantly clear that a phylogenetically reliable and systematically quality-controlled database is needed to serve as a standard for the comparison of any newly reported data whether medical, forensic, or anthropological [7].

The Genographic Project, begun in 2005, allows any individual to participate by purchasing a buccal swab kit. Male samples are analyzed for a combination of male specific Y chromosome (MSY) short tandem repeat loci and SNPs. Female samples undergo a standard mtDNA genotyping process that includes direct sequencing of the extended HVS-I (16024–16569) and the typing of a panel of 22 coding-region biallelic sites. Results are returned anonymously through the Internet (http://www.nationalgeographic.com/genographic) after passing a multi-layered quality check process in which phylogenetic principles are applied throughout, and which is supported by a specialized laboratory information management system. HVS-I haplotypes are reported based on the direct sequencing results. Hgs are defined by a combined use of the 22-SNP panel results and the HVS-I haplotypes. Following successful typing and reporting of the genotyping results, each participant may elect to donate his or her anonymous genotyping results to Genographic's research database. The magnitude of the project and its worldwide scale offer a unique opportunity to create a large, rapidly expanding, standardized database of HVS-I haplotypes and corresponding coding-region SNPs. Here, we report our experience from genotyping 78,590 public participants' mtDNAs during the first 18 months of the project. First, we describe our genotyping process and quality check measures and our considerations in designing them. Second, we report the unique insights that the standardized database supports with respect to estimation of the frequencies of transversions, transitions, heteroplasmies, indels, back mutations, and homoplasy occurring in both the HVS-I and the coding-region biallelic sites. Third, we present a new nearest neighbor (NN) –based methodology developed for Hg labeling, suggest it as an Hg prediction tool for validation of both new and previously reported databases, and demonstrate its superior performance over rule-based approaches, given a sufficiently large reference database. Finally, we make available to the scientific community and general public two new resources: a database (which will be periodically updated) containing the data donated by participants as an open source research database, and the NN analytical tool, which allows the comparison of any comparable data to the entire expanding Genographic dataset for quality control and predictive purposes.

## Results

A total of 78,590 mtDNA samples were analyzed, of which 41,552; 5,046; 15,021; and 16,971, respectively, were genotyped with a panel of 10, 20, 21, and 22 SNPs. We excluded from our analysis samples in which the SNP genotyping result was summarized as “uninformative” and heteroplasmic positions. Therefore, we consider three different versions of the database: (1) The *entire* database: 76,638 samples. (2) The *reference* database made up of the subset of samples genotyped with the panel of 22 SNPs, currently comprising 16,609 samples. This reference database is expanding, as all new samples are genotyped with these 22 SNPs. (3) The *consented* database, released to the public with the participants' consent. So far, data from 21,141 samples (7,174 of which belong to the reference database) have been donated to the scientific community and are reported in Dataset S1 (for future updates of the database please see: http://www.nationalgeographic.com/genographic). Analyses using complete haplotype information are restricted to this dataset. The database presents the following information about each sample: a sequential serial number (different from the anonymous Genographic participant ID number), the number of SNPs genotyped, results of all genotyped SNPs, the Hg inferred from the SNP genotyping, the final Hg assigned in the current study, and the HVS-I haplotype.

### Genotyping Parameters

The genotyping parameters associated with the reference database are presented in Table 1. The overall first pass genotyping success rate for the entire process including DNA extraction, sequencing, and SNP genotyping was 98.5%. The average time needed to complete the first genotyping attempt was 31 days. All samples were attempted with bidirectional sequencing, but 13.9% contained the transition T16189C, which blocks the sequencing reaction beyond this position, and these provided data from only one strand. Of the remaining 86.1% reported samples, forward, backward, and bidirectional sequencing were successful in 99.7%, 99.7%, and 99.4% of the samples, respectively. The alternative forward sequencing primer (Table S1) was used once, while the use of an alternative reverse sequencing primer was mandated in approximately 0.15% of the samples. A total of 83.2% of the samples was successfully genotyped for the complete panel of 22 SNPs. The success rate of inferring an Hg by this SNP panel was 94.7%, while 3.2% and 2.1% of the samples were labeled as inconsistent or uninformative, respectively. The total number of samples from project inception in which post-DNA-extraction sample mix-up was suspected due to clear nonconcordance between Hg labeling, as suggested by the HVS-I motif and the SNP genotyping, was 19 (0.00024%). The total number of samples from project inception in which the genotyping process could not be completed after attempting genotyping from both buccal swabs provided by the participant was 21 (0.00027%).

**Table 1:**
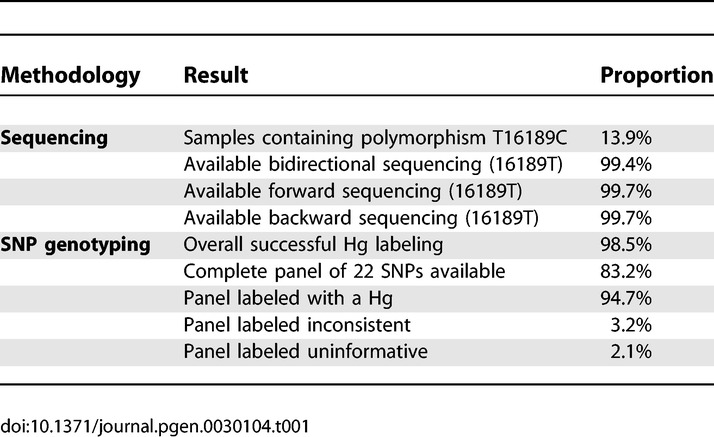
Genotyping Parameters of the Reference Database

### General Indices

Hg frequencies observed in the entire database, the reference database, and the consented dataset of 21,141 records are given in Table 2. In the entire database, the most frequent Hg was Hg H (38.2%). When the database was collapsed into macro Hgs L(xM,N) M, and N the following frequencies were observed, respectively, 4.54%, 3.42%, and 92.04%. Table S4 provides the observed transitions, tranversions, insertions, and deletions for the entire database and further delineates their frequencies within each Hg for the reference database. Note that inferences regarding the number of times each mutation occurred within each Hg are impossible to determine from this table. The total numbers of distinct transitions and tranversions observed were 343 and 199, respectively. The total numbers of distinct insertions and deletions observed were 35 and 15, respectively. Table S5 describes, for the entire database, the number of distinct heteroplasmies observed and further delineates within the reference database their distribution within each Hg. The total number of distinct heteroplasmies was 152. As it is difficult to establish the threshold of heteroplasmy detection by direct sequencing with current technologies, it is likely that the heteroplasmies found are an underestimate [16].

**Table 2:**
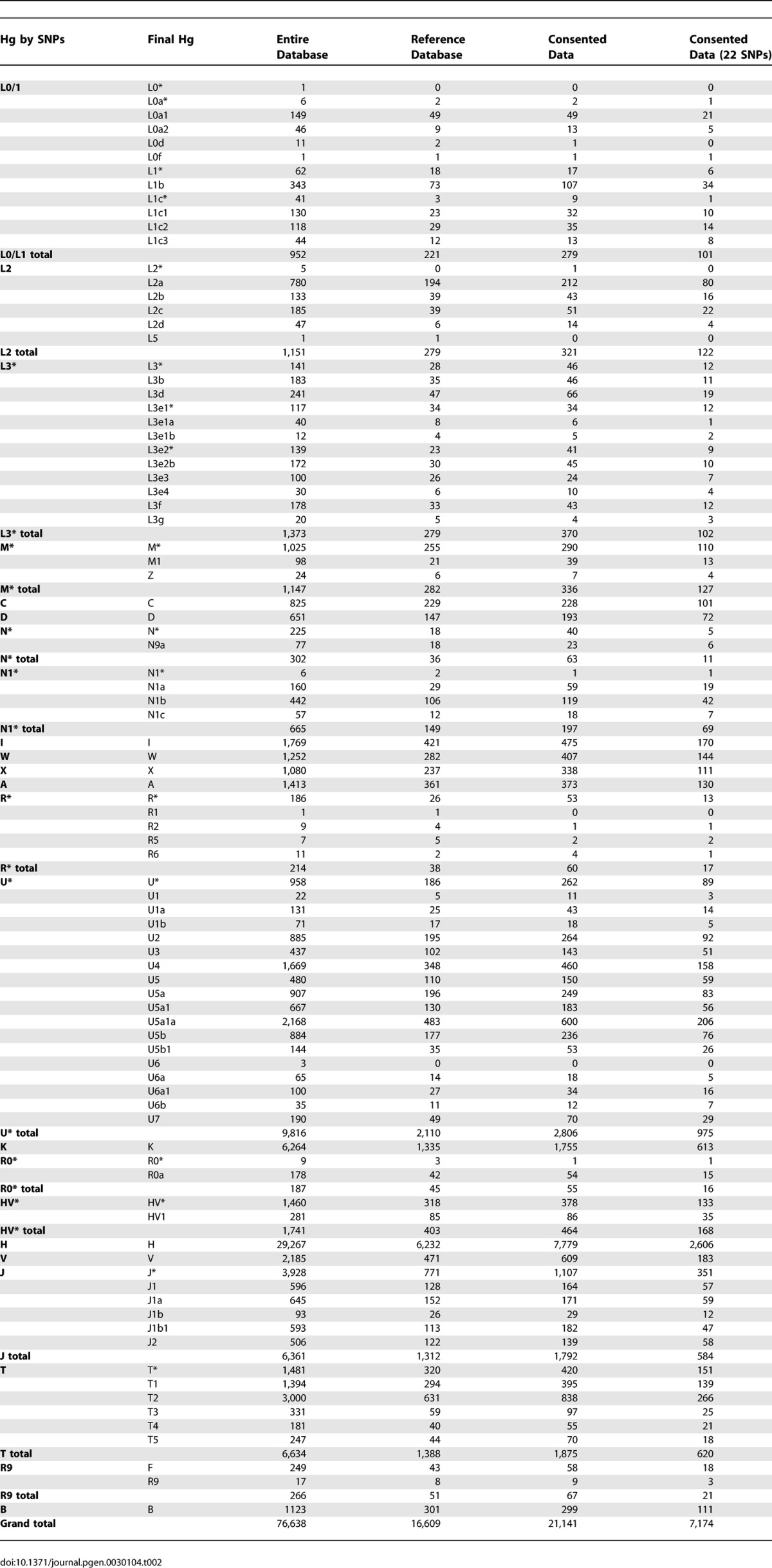
Hg Frequencies

### Homoplasy and Back Mutations in HVS-I Haplotypes

**Figure 1:**
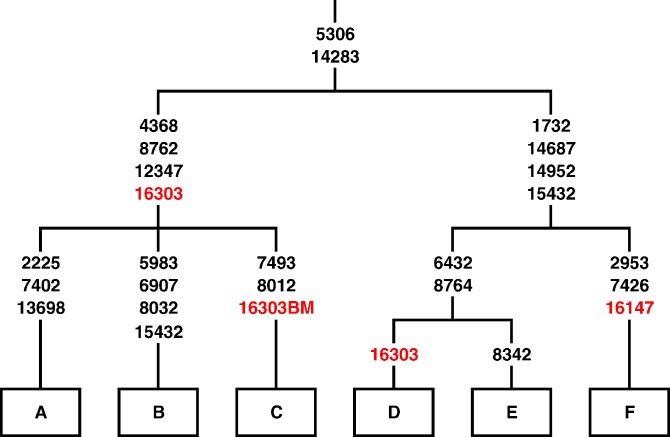
HVS-I Identity by Descent or by State A theoretically evolving tree is presented. Coding-region polymorphisms are in black. HVS-I polymorphisms are in red. Samples A and B share HVS-I haplotype 16303 by descent. Samples A and D or B and D share HVS-I haplotype 16303 by state and as a result of homoplasy. Samples C and E are identical by state as a result of a back mutation in position 16303 in sample C as marked by the “BM” designation.

The results described in this section are from the reference database to provide maximum phylogenetic resolution. Homoplasy is the phenomenon in which the same mutation is found in two distinct phylogenetic branches of the mtDNA tree. Back mutation is defined herein as the phenomenon by which a position considered characteristic or diagnostic to a certain Hg has reverted to the ancestral state. It is clear that the phenomenon can affect any other position as well. The result of both phenomena can be haplotypes that are identical by state but not by descent (Figure 1), and can therefore bias interpretation of databases that make use of HVS-I haplotypes alone to infer Hg labeling or shared ancestry. In addition, these phenomena can also lead to an underestimation of population genetic distances. The extensive database presented herein contains numerous examples of these phenomena, of which many are well known while others are previously unreported. Table S6 shows the number of times that all classic HVS-I Hg-defining mutations are present as part of the haplotype motif in all reported Hgs. Table S7 shows the number of times that the same haplotype occurs in different Hgs for the portion of the reference database that overlaps with the consented database. Table S8 shows the number of times that a sample was assigned to an Hg by the SNP genotype, but did not harbor the classic HVS-I motif as defined in Table S2. Unfortunately, the scope of this paper is too limited to describe all examples and, therefore, we focus on a few examples that emphasize the magnitude of these phenomena.

The haplotype that shows no polymorphic changes when compared to the revised Cambridge Reference Sequence (rCRS) was well-reported under Hgs R*, U*, HV*, H, V, and their sub-branches [2,5,17,18]. These Hgs, which are frequent in populations of European ancestry, are expected to be frequent among the project's largely North American participants. Indeed, our database can be considered to contain an extensive sampling of European-derived populations. Since it is hard to decide whether the HVS-I haplotype of the ancestor of Hg R was rCRS or 16519C when mtDNA positions 16024–16569 are considered, the existence of the former in these Hgs can either represent identity by descent or identity by state due to homoplasy (Figure 1). Whether identical by descent or by state, the use of the 22-SNP panel allows the accurate placing of each mtDNA genome into a single Hg. Of a total of 463 mtDNA genome sequences that contain the rCRS (16024–16569) as their HVS-I haplotype, 416, 26, 1, and 20 would have been assigned to Hgs H, HV*, U*, and V, respectively, when typed with our 22-SNP panel (Table S6). Likewise, any study of a European population that used HVS-I information only and labeled all rCRS samples as identical or as Hg H, is likely to incorrectly assign about 10% of these samples. The large size of the database allows us to estimate the frequencies of additional examples of homoplasy that were previously reported in the literature. Positions 16343G, 16356C, and 16270T are considered characteristic of Hgs U3, U4, and U5, respectively. These positions actually occur in Hg H in 0.7%, 0.1%, and 3.9% of the cases, respectively. Positions 16224C and 16311C are widely considered to characterize Hg K. Our database, however, shows both a branch within Hg H that carries haplotype 16224C–16278T–16293G–16311C and branches within Hg K that lack positions 16224C or 16311C. The characteristic positions for Hg J and T are 16069T–16126C and 16126C–16294T, respectively. Several samples in our database shared the haplotype 16069T–16126C–16294T that contains both characteristic positions and proved to belong to Hg J. Haplotype 16223T–16519C occurred within Hgs H, M*, N*, U*, and W. More complex haplotypes, such as 16223T–16355T–16519C, occurred under both L3* and M*. Haplotype 16223T–16295T–16519C occurred in Hgs M* and W. The combination of positions 16189C–16217C occurred under both Hg B5 and N*. The important branching point between macroHg N and its daughter, macroHg R, is marked by two transitions, T12705C and T16223C. Our database shows that 2.5% of all preHg R mtDNA genomes have lost polymorphism 16223T and 1.1% of all R mtDNA genomes gained this mutation, mostly in the K1a1b1a lineage [19]. More specifically (Table S8), Hg I is characterized by HVS-I positions 16129A–16223T–16391A. Of the 421 Hg I mtDNA genomes defined by the relevant coding-region SNPs, 1.2%, 1.0%, and 3.3% have lost positions 16129A, 16223T, or 16391A, respectively. Of the 282 Hg W mtDNA genomes defined by the relevant coding-region SNPs, 1.4% and 15.2% have lost positions 16223T and 16292T, respectively. Of the 229 Hg C mtDNA genomes defined by the relevant coding-region SNPs, 2.2%,, 2.6% and 0.4% have lost positions 16223T, 16298C, and 16327T, respectively. These examples of positions that have experienced back mutation cannot indicate the number of times that each position has reverted during the Hg's evolution, as within-Hg resolution is not part of the presented database.

### Homoplasy and Back Mutations in Coding-Region SNPs

The results presented below are from the reference database to provide the maximal phylogenetic resolution. Coding-region SNPs, used as reliable markers to define Hgs because they are considered stable evolutionary events, are nevertheless not entirely stable [19–21]. The dataset reported here supports this notion, and the portion of samples in which the SNP genotyping results were shown to be “inconsistent” with the expected phylogenetic hierarchy provides an important opportunity to estimate the extent of this phenomenon. Table S9 gives the number of times each of the tested SNPs occurs in different branches of the phylogeny. The overall frequency of samples in which inconsistency was observed was 3.2%. We note that excluding the 9-bp deletion at position 8280 would decrease the frequency to 2.0%. We highlight a few examples here.

The most trivial is the occurrence of transition A13263G (which we use to identify Hg C) in Hg W. The phylogeny supported by the remaining panel of 21 SNPs correctly places the samples as belonging to Hg W. The occurrence of this transition under Hg W actually defines the samples as belonging to sub-Hg W3 [22]. Hg H, descending from R0, is expected to harbor transitions A11719G, T14766C, and T7028C. However, 83 of the total 6232 Hg H samples lack transition A11719G, of which 73 share the HVS-I position 16316G. The phylogeny supported by the remaining panel of 21 SNPs correctly places the samples as belonging to Hg H, with this subset probably representing a monophyletic clade characterized by the loss of transition A11719G and gain of position 16316G that has not yet been named. An interesting issue concerns transition T12705C, which is the only coding-region mutation known to separate Hgs R and N [21]. Three samples (0.1%) out of the 2,923 that were labeled by the remaining 21-SNP panel to be pre-R lineages did carry this transition, all of which were in Hg L sub-branches. Conversely, a total of 13,686 samples were labeled by the remaining panel of 21 SNPs to be lineages within Hg R, of which seven (0.05%) did not carry transition T12705C (but all carried SNPs typical of Hgs within R). These findings emphasize the importance of this position as separating Hg N from R.

### The NN Methodology

To quantify the effectiveness of the NN/weighted NN (w-NN) method combined with our reference database in mtDNA classification, we tested our ability to recover the classification revealed by the coding-region SNPs in the Genographic database. We consider classification into 23 basal Hgs based on our most extensive SNP typing protocol (22 coding-region SNPs) as a “gold standard” classification (correct with a very high probability), and use it for comparison of the performance of our rule-based and w-NN classification approaches, when classifying based on HVS-I information only (without using the SNPs for classification). For this purpose, we adopted a leave-one-out cross-validation approach, i.e., each of the 16,609 samples for which we have 22 SNPs was left out, and the 16,608 remaining samples were used as a “reference” database for NN/w-NN. The accuracy obtained for recovering the coding-region Hg assignment by the NN/w-NN approaches was 96.72% and 96.73%, respectively (Table S10, last row). While this difference is tiny, we see consistently throughout Table S10 that w-NN does slightly better than NN (win-loss-tie ratio of 35-4-5). We also applied the rule-based approach (Table S2) based on HVS-I only, and obtained an accuracy of 85.3% (Table S10). Our conclusion from this experiment is that the NN-based approaches can support much higher accuracy in classification of our samples (and samples coming from similar populations) based on HVS-I only, when utilizing the Genographic database as reference. Table S10 details the results of repeating the same experiment with a variable number of SNP panels.

### Saturation

**Figure 2:**
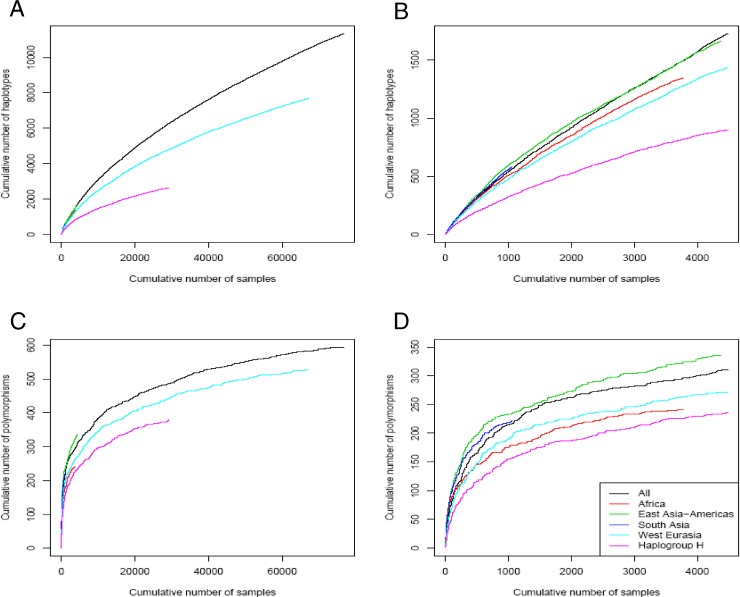
Saturation Curves The number of accumulated mtDNA HVS-I haplotypes (A and B) and polymorphic sites (C and D) as a function of the number of accumulating samples is shown. The analysis is presented once for the entire database (A and C) and once for a limited number of samples (B and D), allowing a better comparison with the less well-represented geographic groups. The Hgs were grossly divided to represent four different geographic groups as follows. Africa: L, M1, and U6; East Asia-Americas: A, B, C, D, F, N9a, and R9; South Asia: M*, R1, R2, R5, and R6; and West Eurasia: N1, R, W, and X. Saturation curves for Hg H are also presented.

We studied the level of haplotype saturation with respect to different HVS-I haplotypes and polymorphic sites present in the database by randomizing the order of the samples in the entire database and plotting the number of newly observed HVS-I haplotypes as a function of the accumulated number of samples (Figure 2). We repeated our analysis for the subsets of Hgs known to represent typically African, West Eurasian, East Asia-Americas, and South Asian mtDNA gene pools, and for Hg H haplotypes. Next, we repeated the analysis for the number of polymorphic sites obtained as a function of accumulated number of samples for the same categories.

The entire database of 76,638 samples was included in this analysis, within which 29,267 belonged to Hg H. A total of 11,346 HVS-I haplotypes were observed in this set (Table S3 shows the partial list of the observed haplotypes in the consented database). Note that homoplasy among these haplotypes is ignored, and the total number of phylogenetically independent haplotypes would have been higher if Hg information had been considered. Figure 2A shows the obtained results for all the haplotypes (11,346), for the groups of Hgs grossly affiliated with Africa (1,348), East Asia-Americas (1,663), South Asia (583), West Eurasia (7,684), and Hg H (2,637). Hgs in which geographic affiliation is uncertain (N*, R*) were excluded from the analysis. Figure 2B repeats the analysis for a limited number of samples to allow better comparison with the less-represented geographic groups. Figure 2C and 2D shows the application of the same analysis to the observed HVS-I polymorphic sites.

### Searching for Evidence of Neanderthal mtDNA and Recombination

We have utilized our database to search for evidence of Neanderthal origin for any of the samples, and for any discrepancies that might be attributed to recombination.

On the Neanderthal question, we first extracted from GenBank all six Neanderthal HVS-I sequences of length at least 300 bp (Table S11). It is now accepted that a combination of five HVS-I mutations (16037G, 16139t, 16244A, 16262T, and 16263.1A), which appears in all of these samples, distinguishes these Neanderthal sequences from modern humans [23]. While all of these five mutations have in fact been observed in our full database of 78,590 samples, no combination of any two of them has appeared in any sample. However, since these six samples may not represent the full diversity of Neanderthal lineages, we have also investigated separately the level of divergence they show from our entire database. No sample in our database is as divergent as these Neanderthal samples, in terms of its distance from its nearest neighbor outside its own Hg, or its distance from the rCRS, which we take to represent a “random” modern human mtDNA (Table S11). We also observe that the most divergent samples in our database all carry well-known HVS-I motifs characteristic of African Hg L branches. While it is difficult to translate these findings into probabilities, it is clear that our results do not support the existence of mtDNA samples of Neanderthal (or other archaic *Homo*) origin in our database.

In the search for recombination, we concentrated on our reference database. If there was a detectable level of recombination in mtDNA, it should lead to phylogenetic inconsistencies in the 22-SNP genotypes. For example, if there was recombination between an Hg H mtDNA and an Hg M mtDNA, where the M sample “donated” its region between nucleotides 9000 and 12000 into the Hg H sample, then positions 10400 (Hg M), 10873 (Hg N), and 11719 (R0), which are in this region and hence in their non-rCRS state, should be “inconsistent” with positions 7028 (Hg H), 14766 (Hg HV), and 12705 (Hg R), which are in their rCRS state. Thus, we extracted all the samples in our reference database that were “inconsistent” (a total of 538 records). Of these, 521 can be explained by a single inconsistency, which can be attributed to a single repeated/back mutation rather than recombination. The remaining 17 require two repeated mutations to explain them. Nine of these 17 cannot be explained by a single recombination event. The remaining eight fall under the Hg H branch, described above, which is marked by the back mutation at position 11719 and by the HVS-I transition A16316G. The second inconsistency in seven of these samples involves the 9-bp deletion at position 8280 and the eighth sample involves an inconsistency in position 13368 (Hg T). As all eight occurred under a phylogenetically consistent branch, we attribute them to repeated mutation rather than a recombination event. We thus conclude that we can find no evidence of recombination in our reference database.

## Discussion

The Genographic Project allows members of the public to participate in a real-time anthropological genetic study. Since its inception in early 2005, over 188,000 individuals have joined the project, of which over 55,000 have submitted their mtDNA or MSY results to the research effort, illustrating the high level of interest. Because any member of the public may participate in the study, rigorously controlled group affiliation data will not typically be obtained for the samples. Furthermore, it is clear that the accumulated database is biased towards countries in which the project is well known and where the kits are economically accessible to a significant fraction of the population. The fact that 95% of the participation kits were ordered in the US and Western Europe is consistent with the Hg frequencies observed, and suggests that the majority of the participants are of West Eurasian (probably European) ancestry.

The importance of improving the quality of the global shared mtDNA database was recently reemphasized and summarized by Bandelt et al [24]. The strict uniform adherence to standard analytic and genotyping protocols across tens of thousands of samples makes the current study an ideal resource for the scientific community. We tried to consider all previously identified sources of errors while designing our genotyping, analysis, and reporting tools. Our database is unique for a project of this scale in using sequencing of both strands of the HVS-I as a standard procedure to assure high-quality data. The same goals led us to incorporate standard coding-region SNP genotyping on all samples. The entire analysis is “pen-less” to avoid any typographic mistakes, and a series of computational quality control measures are embedded in it. Despite the rigorous quality check procedures implemented, we still anticipate some inaccuracies in the database, but believe that these genotyping standards raise the bar on mtDNA genotyping and represent good progress towards more reliable databases.

A few simple measures can be suggested to facilitate future assembly of mtDNA databases. First, as sequencing procedures have become more efficient and stretches of 600 bp can easily be obtained, we suggest standardizing the reported “HVS-I” range to include positions 16024–16569 as presented herein. Second, it would be worthwhile to create a standard list of coding-region SNPs used by the scientific community for Hg assignment and change to alternative coding-region SNPs defining the same Hg when there is a reason to suspect that the standard SNP is misleading due to homoplasy or back mutation. We make available our quality check measures as a model for any future mtDNA database submitted for publication.

The database reported herein is very informative with respect to the mtDNA phylogeny, including the frequencies of the observed haplotypes, transversions, transitions, indels, and heteroplasmic positions both in the coding and control regions (Tables S3–S10). No highly divergent (e.g., Neanderthal) sequences were observed, despite more than doubling the total number of sequences examined, and no evidence for recombination was found. The database did, however, provide evidence for homoplasy and back mutations affecting a low, but not insignificant, percentage of the samples both at the HVS-I and the coding-region SNPs chosen herein. For the coding-region SNPs, even these phenomena do not usually prevent the correct positioning of an mtDNA genome in the phylogeny, as the latter is based on the identification of a string of positions and not a single one. For the HVS-I, our analysis shows that while the use of Hg labeling techniques based on HVS-I variation have an overall good correlation with coding-region SNP genotyping, caution should be used in general, and, in certain specific cases, prediction is best avoided. In population-based studies of large sample size, these phenomena will likely have a small affect on the overall conclusions. However, for individual genotyping, as studied in genealogical or forensic cases, these percentages may be sufficient to preclude, for example, a firm conclusion regarding the time to most common recent ancestor of a set of samples for which only HVS-I information is available.

The NN methodology presented herein, when jointly used with our reference database, has been shown to assign more mtDNA genomes to their correct Hg than prediction methods based on the classic set of HVS-I motifs. Our genotyping strategy, associating each of the HVS-I unique mutations with an Hg confirmed by a coding-region SNP, supplies the needed infrastructure for developing the NN methodology. It is clear that the high prediction score of NN/w-NN is a function of the size of the reference database collected within the population, in which the NN/w-NN methodology is implemented along with the length of the analyzed fragment in the HVS-I. For this study, and considering the large reference database, it was shown that, when no coding-region genotyping was done and Hg prediction was based solely on HVS-I classic Hg-determining rules, as many as 15% of the predictions were wrong, while the w-NN yielded an accuracy of 96.73%. In the sample set studied, the high rates of failure in predicting the correct Hg using HVS-I based rules alone is likely the result of high prevalence of Hgs for which no satisfactory predictive rules exist (such as Hg H and HV*) and to a lesser extent from phenomena like homoplasy or back mutations. To illustrate how the use of the w-NN methodology requires a joint use of a reliable relevant reference database for the studied population, we applied the w-NN methodology and our current reference database to published databases that are external to the Genographic Project and from various populations. West European and non-West Eurasian sequences, the two extremes, yielded prediction scores at a high and a low of 93.8% and 77.9%, respectively (data not shown). Therefore, we make the NN prediction methodologies available on our Web site (http://www.nationalgeographic.com/genographic) in two forms: a) the NN independent code to be used with any reference database and b) in combination with an upload tool that allows the NN methods to be applied to uploaded samples using the Genographic reference database. As emphasized, we expect that the best prediction scores will currently be obtained in samples of West Eurasian ancestry for the 23 basal Hgs defined here, and that the predictions will gradually improve for other populations as the Genographic Project progresses and worldwide samples are obtained and included in the reference database, and as more coding-region SNPs are used to further resolve the basal Hgs into their sub-clades, a process actively underway in the Genographic research consortium.

An interesting question that can be examined using our database relates to the effect of protocols using variable numbers of coding-region SNPs on the accuracy of Hg assignment when compared with the classification of the reference database using the full 22-SNP protocol as a gold standard (as if 100% accurate). Table S10 gives the results for several coding-region SNP protocols of which the 10-, 20-, and 21-SNP protocols were previously used by the Genographic Project. These data show that a high degree of predictive accuracy was rapidly achieved as SNPs were added. When no SNPs were used, the best prediction methodology was with w-NN and yielded an accuracy of 96.73%. The most important single SNP in our population, 7028 (Hg H), allows 98.18% accuracy (w-NN) on its own. The initial panel of ten SNPs, when combined with the HVS-I information, is responsible for 99.81% (w-NN) of the Hg assignment accuracy achieved, and the last 12 SNPs are needed to resolve the remaining small portion of the samples (Table S10).

Our large database allows us to make some simple measurements of haplotype and polymorphic site saturation. Figure 2 shows that even the large number of samples collected in our study does not reach HVS-I haplotype saturation. The discrepancy between the shapes of the haplotype and polymorphic site curves probably means that the number of observed polymorphic sites is closer to saturation than the number of observed haplotypes, which in turn suggests that shuffling of the same polymorphic sites, through homoplasy and back mutations, is the dominant mechanism that increases haplotype variation. These results are not surprising in view of the strong signal of expansion observed in human mtDNA [1]. Indeed, given the huge state space for haplotype motifs, we would expect a large number of haplotypes at very low frequencies, keeping the saturation curve of haplotypes steadily rising. In contrast, the space of sites is tiny, and, therefore, presumably closer to saturation.

**Figure 3:**
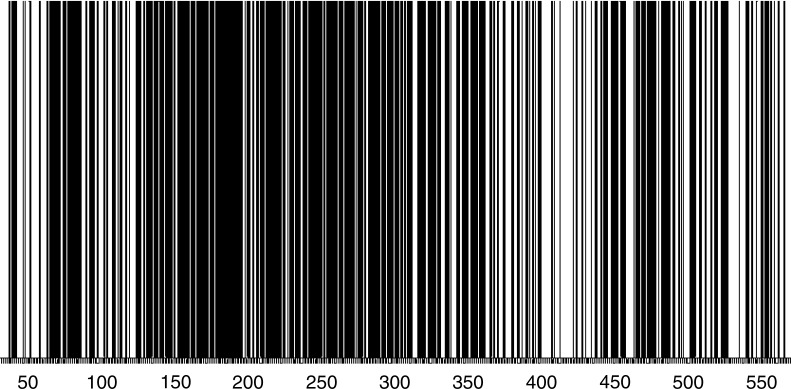
Physical Map of HVS-I The figure presents a simple map made up from all polymorphic sites observed in the sequenced region 16024–16569 without denoting their frequencies. Conclusions regarding the number of times each observed position was hit during *Homo sapiens'* evolution can not be inferred.

The function of the control region is not completely understood, but is thought to be involved in mtDNA genome replication and transcription, and possibly contains the origin of heavy- and light-strand mtDNA replication and several transcription binding sites, with the HVS-I depauperate in regions of this kind [25]. One might expect that the parts of the control region in which these sites are found will be more conserved than others. The information obtained from all unique polymorphic transversions, transitions and indels was used to draw a “bar code” of the sequenced region to show all positions in which a mutation was observed (Figure 3). A total of 358 (65.5%) of the possible 546 sequenced positions showed polymorphism. Some variability in density of polymorphic regions is evident, but no “polymorphism-free” regions can be detected. Note that the map does not distinguish between positions that mutated once or multiple times during the mtDNA evolution of Homo sapiens. It is also important to note that the database does not represent the worldwide variety of mtDNA and, therefore, mutations typical of other populations may not be represented.

A few considerations unique to a public project should be discussed. Because the current dataset presented in this manuscript comprises members of the public who have joined Genographic's research effort, the samples herein represent a subset of the total global mtDNA diversity. To properly survey the genetic variation in non-Western Eurasian lineages, the Genographic Consortium is actively consulting and engaging with members of indigenous communities from around the world, and conducting anthropological and genetic analysis on those DNA samples. As such data are published they will also be made available anonymously as part of the reported Genographic reference database. The classification, saturation, and analytical techniques will need to be updated accordingly, as is the case with any expanding database. In addition, this manuscript presents a level of Hg resolution based on the current 22-SNP panel and HVS-I information. A stated goal of this research effort is to continue to refine and increase this resolution, which will be achieved by further genotyping or revised analysis incorporating the expanding dataset. Therefore, at present, the participants and scientific community are presented with a solid, but still rather simple, level of analytic resolution and are encouraged to return periodically to the project's Web site to access up-to-date data and analytical tools.

In summary, we report both data and new classification methods developed using by far the largest standardized mtDNA database yet created, and detail the logistic, scientific, and public considerations unique to the Genographic Project. Most importantly, we return to the public a database made possible by their enthusiastic participation in the Genographic Project.

## Materials and Methods

### Sampling and sample handling.

The Genographic Project's Web site allows members of the public to order a buccal swab kit (containing two buccal swabs) and undergo genotyping for either mtDNA or MSY analysis. To ensure anonymity, each participation kit is encoded with a randomly generated, nonsequential, Genographic Participant ID number. All samples are genotyped with informed consent according to procedures approved by the Institutional Review Boards of the University of Pennsylvania and the United States Department of Health and Human Services. Once results are obtained, the participants may consent to contribute their genetic data anonymously to the Genographic research database, to be used for anthropological studies and made available to the scientific community. The participants are also asked to provide genealogical information relevant to their deep ancestry.

### Nomenclature.

We use the term haplotype to describe HVS-I variation. The reported HVS-I is “extended” and covers 16024–16569 for all samples. Absolute numbers are used to describe nucleotide position (1–16569) in the mitochondrial genome, and refer to the position of the polymorphism compared with the rCRS [26]. It is common practice to label by letters the nucleotide change only for transversions (e.g., 16318t) and to avoid labeling by letter transitions (e.g., 16093), since the changed nucleotide can be inferred from the rCRS [27]. As this study also addresses the general public, who may not be familiar with rCRS nomenclature conventions, we note here that we deviate from the common practice, and to facilitate reading and the use of the released database we label both. Transitions are labeled by capital letters (16093C), transversions by small letters (16318t), and heteroplasmies by the letter “N” (16189N). Sequencing alignment always prefers 3′ gap placement for indels. Deletions are marked by the letter “D” (16166D) and insertions by the point (.) sign (16188.1C).

We use the term Hg to describe haplotype groups (“haplogroups”) [28] that usually coalesce tens of thousands of years ago and are best defined by a combination of coding-region SNPs. The Hgs currently reported by the Genographic Project are listed in Table 2. We adopt a standard Hg nomenclature scheme [27]. Since we have noted that the asterisk (*) suffix used in this scheme leads to some confusion among public participants, we elaborate here on this point by giving an example. A label such as M* means that a sample belongs to Hg M, but not to any of the known subclades within M. It is temporary, and should mean that the Hg is one of many paraphyletic clades falling under the monophyletic Hg M but is not any of the *known* single-letter (e.g., Hg D) or letter-number (e.g., Hg M1) coded Hg M sub-branches. It is therefore clear that even if all reported databases abided by this definition and labeled M* by excluding all known sub-branches at time of publication, it would be impossible to compare samples that fell into this cluster in different publications, because new sub-branches are continually defined. The solution suggested for Y chromosomal nomenclature [29], which clearly specifies which sub-branches were excluded (e.g., M*(xCZ, M1, M3, M51)), might ease database comparisons, especially, when phylogenetic knowledge enlarges and it becomes harder to exclude all known sub-branches of each given Hg in each study. Therefore, we suggest a slight modification to the use of the asterisk suffix. Herein, its use denotes that the sample was excluded from all sub-Hgs reported in this study only (Table 2), whether defined by a coding-region SNP or an HVS-I defining motif (Table S2). Therefore, in this study, the label M* means that the sample belongs to Hg M and was excluded only from the sub M branches reported in this study; namely, C and D by coding-region SNPs, and M1 and Z by HVS-I defining motifs. The sample could still belong, for example, to the well-defined M5 or M8 branches that are not part of the Hgs reported in this study.

### HVS-I sequencing.

Sequences of an extended HVS-I (16024–16569) are determined from positions 16024 to 16569, by use of the ABI Prism Dye Terminator cycle-sequencing protocols developed by Applied Biosystems (http://www.appliedbiosystems.com). Sequencing is performed on a 3730xl DNA Analyzer (Applied Biosystems). Mutations are scored relative to the rCRS [26]. The primary amplification is achieved by primers 15876F and 639R (Table S1). PCR products are cleaned using magnetic-particle technology (BioSprint 96; Qiagen, http://www.qiagen.com). Following the primary amplifications, all samples are subject to bidirectional sequencing using primers 15946F and 132R (Table S1). In cases of template polymorphism at the annealing site(s) and failed sequencing due to primer/template mismatch, alternative primers are used (Table S1). High quality is assured by the following procedures: (1) All sequences are aligned by the software Sequencher (Gene Codes Corporation) and observed by an operator. (2) All positions with Phred score <30 are directly inspected by an operator [30,31]. (3) All positions that differ from the rCRS are recorded electronically. (4) Forward and backward sequences of all samples are electronically checked for consistency. (5) All scenarios noted herein are highlighted for review: failed samples, inconsistencies in forward and backward sequencing, successful sequencing in one direction only, sequences that contain indels or heteroplasmy, and sequences that are shorter than the required length. (6) All highlighted samples are observed again by a second operator. (7) All sequences containing two or more heteroplasmies are regarded as contaminated and DNA is re-extracted from the second swab of the participant. (8) The list of HVS-I haplotypes observed among the lab staff is presented as part of Dataset S1. (9) All reported variant positions are digitally checked for consistency of the expected order of the mutations (i.e., 16093C followed by 16126C and not 16126C followed by 16093C). (10) All reported variants are verified to represent a real polymorphism by direct comparison to the rCRS. (11) All variants reported for the first time when compared to the entire database are highlighted and re-observed. (12) All data donated to the scientific world with consent are released. Any comments and remarks raised by external investigators after release will be addressed by re-observing the original sequences for accuracy. Following that, any unresolved result will be further examined by re-genotyping and, if necessary, immediately corrected by publishing an erratum.

### Coding-region biallelic site genotyping.

**Figure 4:**
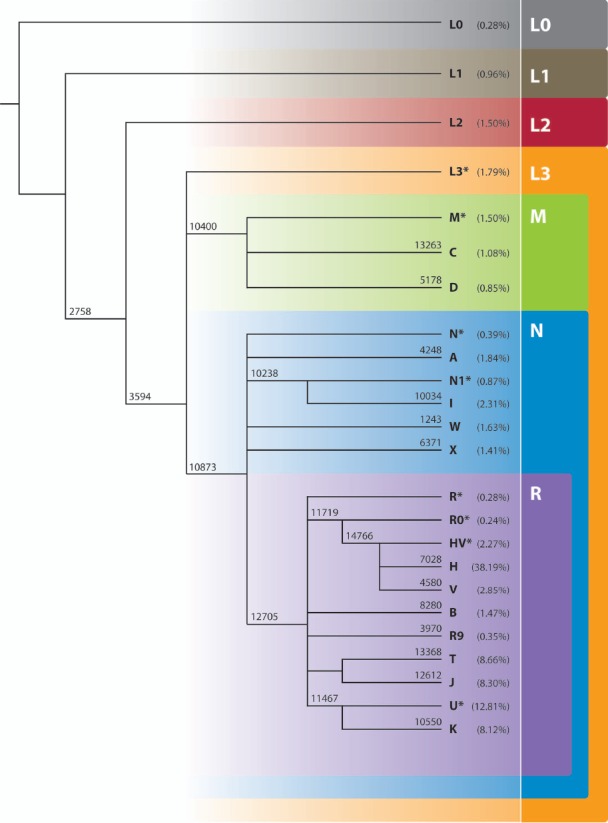
The Phylogeny of mtDNA Haplogroups Inferred from the Panel of 22 Coding-Region SNPs Used in the Genographic Project The coding-region mutations are shown on the branches. The frequencies of the haplogroups found among the Genographic participants are shown in brackets beside the Hgs assignments and correspond to Table 2. Note that the figure discriminates between haplogroups L0 and L1 while the coding-region SNPs used during genotyping do not distinguish the two and therefore they are labeled throughout the paper as L0/L1.

The biallelic sites are genotyped by means of KASPar assays [32] and are independent of the sequencing, thus playing an additional role in the quality check. Twenty one SNPs and the 9-bp deletion make up the total of 22 biallelic sites. For simplicity, we will refer to all biallelic sites as SNPs. The number of SNPs tested was gradually increased from ten at inception of the project to the 22 currently used. The ten initial SNPs were 3594, 4580, 5178, 7028, 10400, 10873, 11467, 11719, 12705, and 14766 (numbers refer to the nucleotide position in the mitochondrial genome). The panel was augmented to a total of 20 coding-region SNPs by including the following additional ten SNPs: 4248, 6371, 8994, 10034, 10238, 10550, 12612, 13263, 13368, and 13928. The panel was further augmented by the addition of SNP 2758, to a total of 21 coding-region SNPs and finally by including the 9-bp deletion at position 8280 to a total of 22 coding-region SNPs (Figure 4). Two further changes were made: positions 8994 and 13928 used in some early work were respectively replaced with their phylogenetic equivalents 1243 and 3970. Therefore, the current panel includes the following SNPs, with their respective gene locations shown in brackets [33]: 2758 (16S), 3594 (ND1), 4248 (M), 4580 (ND2), 5178 (ND2), 6371 (COI), 7028 (COI), 8280 (9-bp deletion) (NC7), 8994 (ATPase6), 10034 (G), 10238 (ND3), 10400 (R), 10550 (NDRL), 10873 (ND4), 11467 (ND4), 11719 (ND4), 12612 (ND5), 12705 (ND5), 13263 (ND5), 13368 (ND5), 13928 (ND5), and 14766 (Cytb). The coding-region SNPs were chosen based on the following considerations: (1) Major branching points in the mtDNA phylogeny obtained using complete mtDNA sequences [21]. (2) Hgs known to be frequent among the current populations in which the project is advertised [2,34,35]. For example, the R0 clade within macroHgs R and N is over-represented. (3) Hgs in which the HVS-I predictive value is known to be unsatisfactory [18]. (4) SNPs reported in previous publications that have been commonly used to identify a particular Hg [2]. For example, we choose polymorphism 7028 and not 2706 to identify Hg H. (5) Technical issues concerning the ability to validate any given assay.

The SNP genotyping results are obtained digitally and analyzed automatically to suggest the appropriate Hg consistent with the mtDNA phylogenetic tree. Two possible scenarios can prevent the reliable assignment of an Hg by SNPs. First, when SNP genotyping in critical positions for labeling a particular Hg has failed due to technical problems, the genotyping result is rendered “uninformative.” Note that most of the information might still exist with only the terminal SNP in the mtDNA phylogeny missing. Second, when SNP genotyping is complete but the reported mutations deviate in a particular SNP from the accepted mtDNA phylogeny, the genotyping result is labeled as “inconsistent” and can result from homoplasy, back mutation, a new unknown SNP next to the checked SNP that distorts the reaction, or a genotyping error.

### Hg assignment.

Hg labeling is achieved by combining the information obtained from (1) the coding-region SNPs, and (2) the HVS-I motifs. A third means of Hg labeling, based on NN methodology, is developed herein.

*Hg assignment by coding-region SNPs.* The standard panel of 22 coding-region SNPs allows a reliable, deep-rooted analysis of the mtDNA phylogeny for each sample as presented in Figure 4. The SNP panel contains a diagnostic SNP for each of the following major bifurcations in the mtDNA phylogeny: L2′3 ′4 ′5 ′6 ′7, L3′4 ′7, M, D, C, N, N1, I, A, W, X, R, R9, B, J, T, U, K, R0, HV, V, and H [1,21]. Therefore, a total of 23 Hg clusters can be inferred from the SNP resolution: L0 or L1, L2 or L5 or L6, L4 or L7 or L3(xM,N), M(xC,D), C, D, N(xN1,A,W,X,R), N1(xI), I, A, W, X, R(xU,R0,J,T,R9,B), U(xK), K, R0(xHV), HV(xH,V), H, V, J, T, R9, and B (Figure 4). To facilitate reading, these 23 Hg clusters are labeled more simply as follows: L0/L1, L2, L3*, M*, C, D, N*, N1*, I, A, W, X, R*, U*, K, R0*, HV*, H, V, J, T, R9, and B. We emphasize that these labels are not equivalent to the final Hg definitions. It is important to note that the use of the coding-region SNPs is very accurate but still prone to errors. For example, under a theoretical scenario in which a sample that belongs to Hg H has a back mutation in position 7028, the panel will label it as HV. We have no way of estimating the frequency with which such a scenario might occur, as we test only one coding-region SNP per branch, but we expect that this phenomenon is very rare.

*Final Hg labeling.* Final resolution to Hgs and sub-Hgs is achieved by comparing and combining the information obtained from the SNP genotyping with the HVS-I motifs. All HVS-I haplotypes obtained following sequencing are digitally screened for possible Hg and sub-Hg definitions by use of accepted HVS-I diagnostic motifs (Table S2) [2,3,36]. The list presents only the motifs used herein for prediction purposes and should not be treated as comprehensive for all Hg suggestions that might rise from HVS-I variation or as representing the Hg basal HVS-I motifs. First, a screen in the *a priori* defined order presented in Table S2 is run and stopped at the first Hg where the sample matches the motif. A second screen for all possible Hgs the sample can fit in is then conducted. It is clear that relying on the HVS-I variation alone to infer Hgs and sub-Hgs such as M1, Z, U5, U6, and HV1 is prone to inaccuracies. In addition, HVS-I haplotypes alone cannot identify Hgs or sub-Hgs that have no defining motifs and ignores the possibility of homoplasy and back mutations. For example, it is clear that some of the mtDNA genomes appearing in our database as U* might actually belong to Hg U4 but, as they did not contain the diagnostic HVS-I position 16356C and in the absence of additional coding-region genotyping, we could not label them as such. Moreover, some of the sub-Hg definitions inferred from the HVS-I, for example within Hgs J and T, will have to be revised in the future as studies using complete mtDNA sequences prove they do not represent monophyletic clades [20,37]. Therefore, whenever analysis is done within an Hg, we refer only to one of the 23 Hgs directly inferred from the 22 SNPs genotyping to avoid any HVS-I based Hg labeling misinterpretations.

Quality checking of Hg labeling is as follows: (1) All discrepancies between HVS-I and SNP labeling are observed by an operator. These discrepancies usually derive from well-known cases of homoplasy and are easily resolved by adhering to the SNP genotyping that correctly assigns the sample to a single Hg in the mtDNA phylogeny. In cases of inability to resolve the discrepancies, the genotyping process is repeated. (2) All samples in which the SNP information is uninformative are observed by an operator. An attempt to label the final Hg is made by direct observation from the partial list of SNPs available and the HVS-I motif. In case of any persisting doubt, the sample is re-genotyped. (3) All samples in which the SNP information is inconsistent are observed by an operator. The Hg assignment is accomplished after studying the entire string of available mutations and by applying the principle of parsimony. The final Hg can be further supported by the HVS-I information.

### Statistical analysis.

*General indices.* Success rates of each of the genotyping processes, Hg frequencies, and distributions including the frequencies of transversions, transitions, heteroplasmies, indels, back mutations, and homoplasy occurring in the HVS-I after taking into account the checked coding-region SNPs are determined by direct counting. We report the heteroplasmic positions in Table S5 but excluded them from all other analyses.

*NN classification methodology.* The common practice of classifying samples into Hgs based on HVS-I information relies on a set of rules that define certain HVS-I backbone haplotypes as characteristic of specific Hgs by using the state of the art knowledge in the literature [2,3,36]. These characteristic motifs, implemented by us here as one of the Hg labeling techniques, are best if previously proven to be associated with particular coding-region SNPs identifying the suggested Hg, and then used to classify newly obtained HVS-I data into Hgs. The weakness of this approach is its sensitivity to phenomena such as homoplasy or back mutations in the motif's HVS-I positions, which may occur between Hgs or within sub-branches of the same Hg. Since parallel evolution is rampant in HVS-I, this issue casts doubt on the ability of rule-based classification to reach high levels of accuracy in certain cases [1,17–19].

Given a large enough “reference” data base of correctly labeled samples (for example, if all samples are verified by coding-region SNPs), we are likely to better assign Hgs for HVS-I haplotypes of new samples if we compare them to all available records in the reference database by identifying their “nearest neighbor,” i.e., the most similar sample we have already classified with confidence. This allows us to use *all of the HVS-I information* in each classification decision, rather than simply counting on the rule-defining sites. Thus, *any* mutations within Hgs that have appeared in the samples in the reference database will be useful for classification, and recent homoplasy in a single HVS-I locus will have a more minor effect on our classification, because other loci within the HVS-I will still support the correct classification.

Given a backbone database D comprising correctly classified HVS-I samples *s_1_,…,s_n_* and a new HVS-I sample *t,* we define the pair-wise distance as *d*(*s_i_, t*) *=* Σ*_j_*_∈*J*_ *w_j_ I*{*t_j_*≠*s_ij_*}, where *J* is the set of HVS-I loci (defined as 16024–16569 in our case), and *w_j_* is a locus-dependent weight. In a simple application (unweighted NN) we would simply take *w_j_ =* 1 ∀*j* and get the (unweighted) Hamming distance, often used in neighbor-joining algorithms. A more reasonable approach would be to down-weigh the loci with a higher mutation rate (such as 16311). Denote these mutation rates (in units of “mutations per year”) as *p_1_,…p_J_*. Then a weighting of *w_j_* = log(20,000 × *p_j_*) can lead to an interpretation of NN Hg classification based on *d*(*s_i_, t*) as an approximate maximum likelihood estimate of the Hg, using the following logic: Assume that the “average” sample has a NN with coalescent time of about 10,000 years. Then the number of mutations separating the sample from its NN in site *i* has a Poisson (20,000 × *p_i_*) distribution, under sufficiently simple substitution models. If we assume that 20,000 × *p_i_* is still very small, as would be the case for practically all sites, then we can approximate the Poisson by a Bernoulli (20 000 × *p_i_*) (which is 1 if the samples differ in site *i*). Now, if we treat the identity of the NN as the parameter to be estimated, we can see that a maximum likelihood estimate would lead us to choose the one minimizing *d*(*s_i_, t*) *=* Σ*_j_*_∈*J*_ *w_j_ I*{*t_j_≠s_ij_*}.

The w-NN analysis requires calculation of site-specific mutation rates, like the ones recently proposed by Bandelt et al. [38] We were limited in our ability to use these published rates, as they only apply to the region 16051–16365, rather than our HVS-I definition. Thus, in our experiments below we use a set of probabilities we derived using a novel methodology (Rosset et al., in preparation). We verified that these estimates are consistent with Bandelt et al. [38] for the region in common, and use them here since they are the only complete set we could obtain. An improved set of probability estimates may improve the results further.

?In applying the NN methodology, we are bound to encounter many “ties,” when there are two equally close NNs in two different Hgs. In our implementation, we assign the new sample to the Hg in which the most similar haplotypes are most prevalent in the reference database.

## Supporting Information

Dataset S1 The Genographic Project Open Resource Mitochondrial DNA Database (Consented Database) (8.1 MB XLS)

Table S1 Amplification and Sequencing Primers (4 KB XLS)

Table S2 Hg-Predicting Motifs (9 KB XLS)

Table S3 Haplotypes Observed in the Database (Consented Database) (457 KB XLS)

Table S4 Polymorphic Sites Observed in the Database (Entire and Reference Database) (189 KB XLS)

Table S5 Heteroplasmic Sites Observed in the Database (Entire and Reference Database) (48 KB XLS)

Table S6 Classic HVS-I Motif Homoplasy (Reference Database) (17 KB XLS)

Table S7 Haplotypes Homoplasy (Consented Database, 22-SNP Group) (745 KB XLS)

Table S8 Classic HVS-I Haplotypes Back Mutation Events (Reference Database) (16 KB XLS)

Table S9 Coding-region SNPs Homoplasy (Reference Database) (8 KB XLS)

Table S10 Hg Prediction by Classic HVS-I Rules and NN Using Variable Sets of Coding-Region SNPs (Entire Database) (10 KB XLS)

Table S11 Divergent Measures of Neanderthals (Entire Database) (4 KB XLS)

Video S1 Introductory Video of the Genographic Project (27 MB MOV)

## Abbreviations

Hg: haplogroup
HVS-I: first hypervariable segment
MSY: male specific Y chromosome
mtDNA: mitochondrial DNA
NN: nearest neighbor
rCRS: revised Cambridge reference sequence
w-NN: weighted nearest neighbor
